# A Comparative Study of the Effect of Anatomical Site on Multiple Differentiation of Adipose-Derived Stem Cells in Rats

**DOI:** 10.3390/cells10092469

**Published:** 2021-09-18

**Authors:** Hanan Hendawy, Masahiro Kaneda, Elsayed Metwally, Kazumi Shimada, Takashi Tanaka, Ryou Tanaka

**Affiliations:** 1 Laboratory of Veterinary Surgery, Tokyo University of Agriculture and Technology, Tokyo 183-8509, Japan; hanan_attia@vet.suez.edu.eg (H.H.); ruiyue1221@gmail.com (K.S.); bamse.rizea.vicky.polta@gmail.com (T.T.); 2 Department of Veterinary Surgery, Faculty of Veterinary Medicine, Suez Canal University, Ismailia 41522, Egypt; 3 Laboratory of Veterinary Anatomy, Division of Animal Life Science, Tokyo University of Agriculture and Technology, Tokyo 183-8509, Japan; kanedam@cc.tuat.ac.jp; 4 Department of Cytology and Histology, Faculty of Veterinary Medicine, Suez Canal University, Ismailia 41522, Egypt; elsayedalalfey@vet.suez.edu.eg

**Keywords:** ASCs, harvest site, flow cytometry, pluripotency, differentiation, rat

## Abstract

Mesenchymal stem cells (MSCs) derived from adipose tissue are evolved into various cell-based regenerative approaches. Adipose-derived stem cells (ASCs) isolated from rats are commonly used in tissue engineering studies. Still, there is a gap in knowledge about how the harvest locations influence and guide cell differentiation. This study aims to investigate how the harvesting site affects stem-cell-specific surface markers expression, pluripotency, and differentiation potential of ASCs in female Sprague Dawley rats. ASCs were extracted from the adipose tissue of the peri-ovarian, peri-renal, and mesenteric depots and were compared in terms of cell morphology. MSCs phenotype was validated by cell surfaces markers using flow cytometry. Moreover, pluripotent gene expression of *Oct4, Nanog*, *Sox2*, *Rex-1*, and *Tert* was evaluated by reverse transcriptase-polymerase chain reaction (RT-PCR). ASCs multipotency was evaluated by specific histological stains, and the results were confirmed by quantitative polymerase chain reaction (RT-qPCR) expression analysis of specific genes. There was a non-significant difference detected in the cell morphology and immunophenotype between different harvesting sites. ASCs from multiple locations were significantly varied in their capacity to differentiate into adipocytes, osteoblastic cells, and chondrocytes. To conclude, depot selection is a critical element that should be considered when using ASCs in tissue-specific cell-based regenerative therapies research.

## 1. Introduction

MSCs are an extensively studied source of cells for regenerative medicine, and they have been a critical cellular component for tissue engineering [1]. MSCs are found in a variety of adult tissues derived from the embryo’s mesodermal layer, such as the bone marrow [2] adipose tissue [3,4], muscle tissue [5], umbilical cord blood [6], periosteum [7], dental pulp [8], exfoliated deciduous teeth [9], periodontal ligament [10] and pancreatic tissues [11].

MSCs have various distinct properties, including the ability to adhere to the plastic, proliferate extensively, express several standard cell surface antigens, and differentiate into multiple lineage-specific cell types [12].

Adipose tissue is a rich and accessible source of adult stem cells with multipotent character, ideal for tissue engineering and regenerative medicine applications. Adipose-derived stem cells (ASCs) are characterized by their adherence to plastic [13]. Furthermore, ASCs are able to differentiate into different cell types [14] such as osteoblasts [15], chondroblasts [16,17], adipocytes [18,19,20], skeletal, smooth and cardiac myocytes [21,22]. Therefore, ASCs are gaining popularity for tissue engineering applications [23]. The anatomical sampling site is still essential for MSC yield and may alter inner cell properties and enhance differentiation [24,25].

The selection of optimal tissue sources of ASCs from various fat depots for cell-based therapy is essential. However, it is still challenging [26]. Although many stem cell sources are available, these do not constantly result in significant growth rate and differentiation potential at the targeted tissues following implantation. Different fat depots contribute differently to disease and function, and these variations could be attributable to regional variances in cell types and fat cell progenitors’ inherent characteristics [27]. Additionally, previous studies showed that MSCs isolated from various origins are not a homogeneous population, and their differentiation capacity varies depending on the source and donor [28,29,30].

In tissue engineering investigations, MSCs generated from rats are widely used. Despite this, considerable differences have been identified across rat MSCs. Furthermore, the knowledge of the effects of anatomical locales on the differentiation potential of ASCs is limited. As a result, the purpose of this research was to determine the impact of the harvesting site on morphology, immunophenotype, pluripotency, and multipotency of ASCs from different locations of visceral fat in the female Sprague Dawley (S.D.) rat body and may be of considerable benefit for tissue engineering applications.

## 2. Materials and Methods

### 2.1. Experimental Animals

Five healthy pathogen-free female S.D. rats at the age of eight weeks old weighing 250–300 g were used in the present study. We selected female rats to obtain peri-ovarian adipose tissue in addition to peri-renal and mesenteric adipose tissue. Rats were housed in cages in a 20–25 °C and 40–60% humidity room, with a 12 h light/dark cycle and plenty of dry food and water. Rat diets were supplied by a commercial company (oriental yeast Co., Ltd., Tokyo, Japan). These diets provide energy and protein levels of 359 Kcal/100 g and 23.1 g/100 g, respectively. Euthanasia of the animals was performed with the inhalation method (isoflurane over-dose), according to Siennicka et al. [31].

### 2.2. Rat ASCs Isolation

Adipose tissue was collected from rat visceral fat of three anatomical locations (peri-ovarian, peri-renal, and mesenteric regions) under sterile conditions. The collected adipose tissue was extensively washed with PBS, placed in a sterile culture plate in a safety cabinet, and minced using sterile scissors. Then, minced adipose tissue was placed in a shaking bath for 1 h at 37 °C in Hank’s balanced salt solution (HBSS) containing 0.1% (*w*/*v*) collagenase type 1 (1 mg/mL; Gibco by Life Technologies, Waltham, MA, USA). Cold HBSS was added to neutralize the collagenase enzyme activity. Following centrifugation (800× *g*, 10 min), we obtained a high-density cell pellet. Solid aggregates were removed via filtration through a 100 μm filter (BD Falcon, Bedford, MA, USA). A 1 mL lysis buffer (Promega, Mannheim, Germany) was used to resuspend the pellet and lyse RBCs. Finally, the cell pellet was resuspended in Dulbecco’s Modified Eagle Medium (DMEM), containing 4500 mg/L D-glucose, 110 mg/L Sodium Pyruvate, 4 mM L-Glutamine, 10% Fetal Bovine Serum (FBS), 1% non-essential amino acids, and 1% Penicillin-Streptomycin (P/S), as a basal culture medium [32]. Unless otherwise specified, we purchased all chemicals from Sigma-Aldrich (St. Louis, MO, USA).

### 2.3. In Vitro Culture of Rat ASCs

Cells were cultured in 75 cm^2^ culture dishes (Nunclon, Thermo Scientific, Waltham, MA, USA), 5 × 10^5^ cells per dish, a humidified incubator at (37 °C, 95% humidity, and 5% CO_2_). Cells were fed with a new medium every three days until reaching 70–80% confluence. Cultures were trypsinized (0.25% trypsin, 37 °C) and resuspended in DMEM +10% FBS before being transferred to fresh culture dishes when they reached 80% confluence. Cells at passage three were used in all experiments.

### 2.4. Evaluation of Morphology and Immunophenotypic Characterization of rat ASCs (3rd Passage)

Cell morphology was assessed using an optical microscope (Cell Sens Standard; Olympus; Tokyo, Japan), and cell shapes were evaluated [33]. The MSCs surface markers cluster of differentiation (CD90, CD44, CD29) and the CD45 hematopoietic marker were analyzed using flow cytometry as previously designated [33]. Cells at passage three were isolated using trypsin. After total cell number was counted, cell suspensions were stained with the following phycoerythrin (PE) fluorescence-conjugated antibodies: anti-CD90 (1:100; cat. No. 551401; BD Biosciences, San Diego, CA, USA), anti-CD44 (1:100; cat. No. ab23396; Abcam, Inc., Cambridge, UK), anti-CD29 (1:100; cat. No. 562154; BD Biosciences, San Diego, CA, USA) and anti-CD45 (1:100; cat. No. 202207; BioLegend, Inc., San Diego, CA, USA) for 30 min at 4 °C.

The CytoFLEX flow cytometer (Beckman Coulter, Brea, CA, USA) equipped with a blue laser (488 nm) was used to assess surface markers (CD90, CD44, CD29, and CD45) expression.

### 2.5. Pluripotent Gene Expression of Cultured Rat ASCs

Reverse-transcriptase PCR (RT-PCR) was used to assess the expression of the pluripotent-related genes, Octamer-binding transcription factor 4 (*Oct4*), Embryonic stem cell-specific homeobox protein (*Nanog*), SRYbox containing gene 2 (*Sox2*), Reduced expression 1 (*Rex-1*) genes, and Telomerase Reverse Transcriptase (*Tert*). Total RNA from rat ASCs of the 3rd passage from different harvesting sites was isolated using the FastGene RNA Premium Kit (NIPPON Genetics, Tokyo, Japan). RNA quantity and quality were measured by a NanoDrop 2000 ultra-micro spectrophotometer (Thermo Fisher Scientific, Cambridge, MA, USA). A total of 1 µg of RNA (OD260/OD280 ≈ 2.0) was reverse transcribed using the PrimeScript RT reagent Kit (Takara Bio, Shiga, Japan). The resulting cDNA was then used as a template for PCR amplification.

Primers were designed based on GenBank sequences (*Oct4*: NM_001009178.2, *Nanog:* XM_006237310.3, *Sox2:* NM_001109181.1, *Rex-1*: XM_032907726.1 and *Tert:* NM_053423.1) using Primer3web, version 4.1.0 (https://primer3.ut.ee/, accessed on 7 August 2021) and purchased from FASMAC company, Japan. The designed primers were as follows: *Oct4* forward (5′-CGAACCTGGCTAAGCTTCCA-3′) and reverse (5′-GCCATCCCTCCACAGAACTC-3′) primers, *Nanog* forward (5′-TACCTCAGCCTCCAGCAGAT-3′) and reverse (5′-CATTGGTTTTTCTGCCACCT-3′) primers; *Sox2* forward (5′-CTCGCAGACCTACATGAAC-3′) and reverse (5′-TCGGACTTGACCACAGAG-3′) primers, *Rex-1* forward (5′-GCTCCGGCGGAATCGAGTGG-3′) and reverse (5′-GCACGTGTTGCTTGGCGACC-3′), and *Tert* forward (5′-CCCGAGTATGGCTGCATGAT-3′) and reverse (5′-AAAGTCCGAGTGTCCAGCAG-3′) primers. *β-actin* forward (5′-GCAGGAGTACGATGAGTCCG-3′) and reverse (5′-ACGCAGCTCAGTAACAGTCC-3′) was used as a control for PCR efficiency evaluation. The RT-PCR mixtures comprised 25 µL EmeraldAmp MAX PCR Master Mix (Takara Bio, Shiga, Japan), 2 µL template cDNA, 0.2 µM of each specific forward and reverse primer, and ddH_2_O up to 50 µL. All reactions were run in triplicate. Cycling protocols were as follows: pre-denaturation at 94 °C for 3 min; 35 cycles of denaturation at 94 °C for 30 s; annealing at 60 °C for 30 s; and extension at 72 °C for 1 min; and final extension at 72 °C for 5 min. All reactions were performed in a Veriti Thermal Cycler (ThermoFisher Scientific, Waltham, MA, USA). PCR products were visualized by gel electrophoresis in 2% agarose (Promega, Madison, WI, USA) in a TBE buffer (Invitrogen) and stained with GelGreen Nucleic Acid Gel Stain (Biotium, Fremont, CA, USA).

### 2.6. Rat ASCs Differentiation (Adipogenesis, Osteogenesis, and Chondrogenesis)

We used a modified version of Lotfy et al. [34] protocol in this study. Plastic-adherent ASCs at the third passage were evaluated for adipogenic, osteogenic, and chondrogenic differentiation capacity. ASCs in DMEM+10% FBS at a density of 10^5^ cells per well were used as a control.

#### 2.6.1. In Vitro Osteogenic Induction

In a 6-well plate, ASCs at the 3rd passage were counted and seeded at a density of 5 × 10^4^ per well. On reaching 80% confluence, osteogenesis differentiation medium (DMEM supplemented with 10% FBS, 0.1 μM dexamethasone, 50 mΜ ascorbic acid, and 10 mM *β*-glycerol phosphate) was added to four wells. The other two wells were supplemented with an uninduced culture medium as negative controls, and the medium was renewed every three days for 21 days. Alizarin red stain (ALZ) evaluated the osteogenesis differentiation capability by visualizing the mineralized matrix. Cells were fixed with 4% (*v*/*v*) paraformaldehyde for 20 min and then rinsed with PBS. After that, the ALZ staining was performed at pH 1. All steps were repeated using ASCs from the three different harvesting sites.

#### 2.6.2. In Vitro Chondrogenic Induction

The third passage ASCs from various sites were counted and seeded 5 × 10^4^ per well in a serum-free cell culture medium for chondrogenic lineage development (Promo Cell, Cat.No.C-28012). Over 21 days, the medium was changed every three days. We used the Alcian blue staining to evaluate chondrogenesis under a light microscope. Alcian blue stain can stain the highly sulfated proteoglycans found in cartilaginous matrices.

#### 2.6.3. In Vitro Adipogenic Induction

For each harvesting site, ASCs at the 3rd passage were counted and seeded (10 × 10^4^ cell/well) in a 6-well plate. At 100% ASCs confluence, an adipogenic-induction medium was added to four wells. This medium was formed from DMEM supplemented with 10% FBS, 1 μM dexamethasone, 500 μM isobutylmethylxanthine (IBMX), 200 μM indomethacin, and 5 μg/mL insulin. However, an uninduced culture medium (ASCs in DMEM+10% FBS only) was introduced into the two empty wells as a negative control. The media were changed in all wells every three days. We collected cells after eight days and used the Oil Red O stain to detect intracellular lipid accumulation.

### 2.7. Reverse Transcription-Quantitative Polymerase Chain Reaction (RT-qPCR)

Total RNA was extracted from the differentiated ASCs using the FastGene RNA Premium Kit (NIPPON Genetics, Tokyo, Japan) according to the manufacturer’s instructions. RNA quantity and purity (OD260/OD280 ≈ 2.0) were evaluated by a NanoDrop 2000 ultra-micro spectrophotometer (Thermo Fisher Scientific, Cambridge, MA, USA). The first-strand cDNA was synthesized from 1 µg RNA using a reverse transcription kit, PrimeScript RT reagent Kit (Takara Bio, Shiga, Japan), according to the manufacturer’s instructions. Relative quantification of the expression of pluripotent related genes was detected using StepOnePlus™ Real-Time PCR System (Thermo Fisher Scientific, Waltham, MA, USA). The primers sequence of adipogenic specific genes; Complement factor D (*CFD*) and Adiponectin (*ADIPOQ*); osteogenic specific genes, Bone Morphogenetic Protein 2 *(BMP2*), Osteopontin (*OPN*), Bone Sialoprotein (*BSP*), and chondrogenic specific genes, Aggrecan (*ACAN*), Collagen type 2 (*COL2A1*), and Chondromodulin-1 (*CHM1*), are listed in Table 1. The qPCR system comprised 1 μL cDNA, 0.5 μL of each forward and reverse primer (10 μmol/L), 10 μL THUNDERBIRD^®^ Next SYBR^®^ qPCR Mix (TOYOBO Life Science, Osaka, Japan), and 8 μL ddH_2_O. The thermocycling conditions for qPCR were 95 °C for 30 s, followed by 40 amplification cycles of 95 °C for 5 s and 60 °C for 30 s. Relative quantification was calculated with the 2^−ΔΔCq^ method [35] and normalized to *β*-actin. Data are presented as expression levels relative to the expression level in the control cells. 

### 2.8. Statical Analysis

All data were presented as mean ± standard deviation (SD). The GraphPad Prism software version 6 (GraphPad Software, Inc., La Jolla, CA, USA) was utilized for statistical analysis. The one-way analysis of variance (ANOVA) and Tukey’s post hoc test detected significant differences between data. A *p*-value less than 0.05 indicated statistical significance, and additional significance was indicated with ** *p* < 0.01 and *** *p* < 0.001. Image J, version 1 (National Institutes of Health, Bethesda, MD, USA; http://www.Imagej.nih.gov/ij/ (accessed on 7 August 2021), RRID: SCR 003070) was used to quantify staining intensities.

## 3. Results

### 3.1. Cell Culture

**Figure 1:**
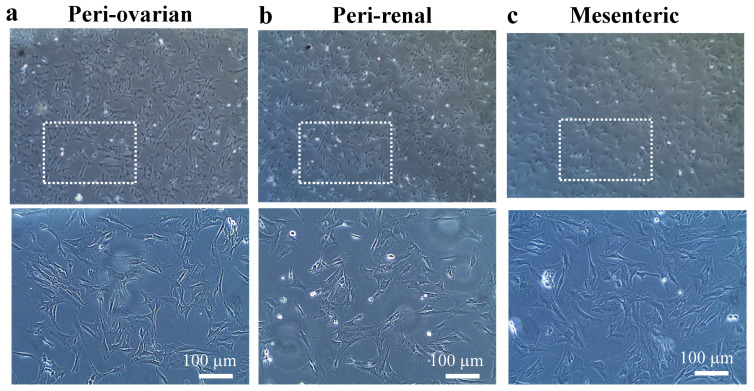
Cell morphology of plastic-adherent ASCs of the 3rd passage derived from enzymatically digested adipose tissue of three different harvesting sites in the female S.D. rat body (**a**) peri-ovarian, (**b**) peri-renal (**c**) mesenteric depots. ASCs at the 3rd passage were characterized based on their fibroblastic appearance. The scale bar represents 100 μM.

ASCs were successfully obtained by enzymatic digestion of fatty tissue at the different harvesting sites. After one day of ASCs culture, spindle-shaped cells were found attached to tissue culture plastic dishes. In 5 to 6 days, primary cultures reached 70 to 80% confluence. The ASCs isolated from all the three sites (peri-ovarian, peri-renal, and mesenteric regions) had a fibroblast-like appearance, with no morphological variations (Figure 1).

### 3.2. Immunophenotypic Characterization of Rat ASCs (3rd Passage)

**Figure 2:**
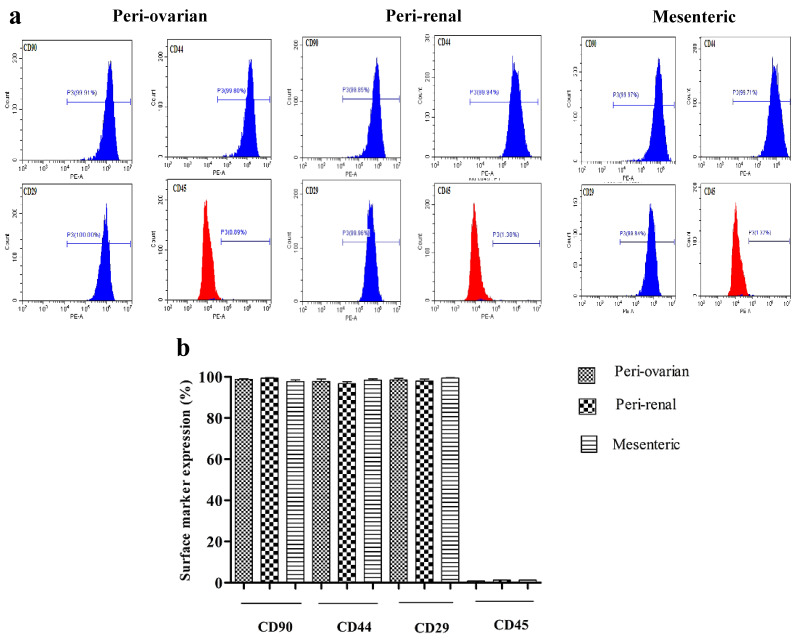
Comparison of the cell surface marker expression of CD90, CD44, CD29, and CD45 of the third passage of in vitro cultures of ASCs from the different sites of female S.D. rats. Cells were incubated with the respective PE-labelled antibodies and analyzed by flow cytometry. (**a**) Representative histograms of each surface marker expression. These cells expressed mesenchymal stem cell-specific markers, including CD90, CD44, and CD29, but showed negative expression to the hematopoietic marker CD45. (**b**) Positive expression for each surface marker. Values are presented as the mean ± SD (*n* = 5) with the one-way analysis of variance (ANOVA) followed by Tukey’s post hoc test. A non-significant difference was detected in the surface marker expression between different harvesting sites.

Cultures of the third passage of ASCs from the different sites were analyzed to express MSCs-specific cell-surface markers (CD90, CD44, CD29) and the hematopoietic marker (CD45). The ASCs population from various harvesting sites shared a standard marker signature of MSCs (CD90, CD44, and CD29 positive expression), with a non-significant difference (Figure 2). However, all cell populations showed negative expression for the hematopoietic marker CD45 with a non-significant change between different sites (Figure 2).

### 3.3. Pluripotent Gene Expression of Rat ASCs

**Figure 3:**
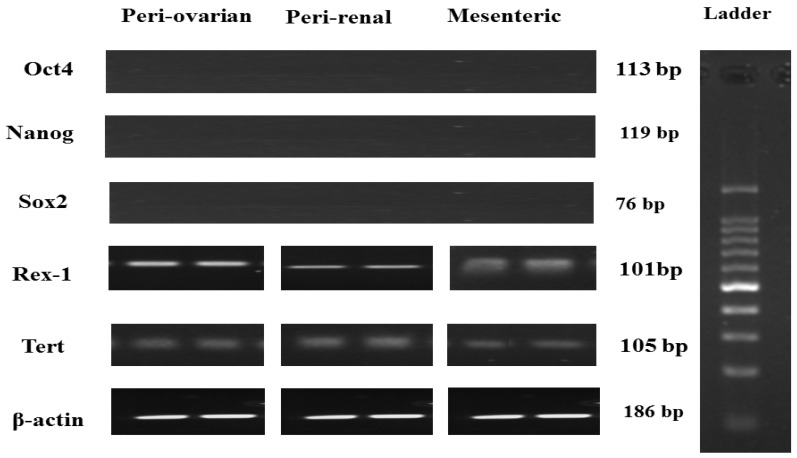
RT-PCR analysis of pluripotency gene expression of female S.D. rat ASCs at the third passage from different harvesting sites. *Oct4*, *Nanog*, and *Sox2* genes were negatively expressed, while *Rex-1* and *Tert* were positively expressed in ASCs from various harvesting sites. *β*-actin served as an internal control.

Specific primers were used for RT-PCR analysis of the pluripotency gene expression indicators, *Oct4*, *Nanog*, *Rex-1*, *Sox-1*, and *Tert*. ASCs of the third passages from the different anatomical sites did not express *Oct4*, *Nanog*, and *Sox2* but showed positive expression of *Rex-1* and *Tert* (Figure 3).

### 3.4. Rat ASCs Differentiation Potential

The multipotentiality capabilities of ASCs from anatomically diverse fat depot sites were studied by growing the undifferentiated cells in specific culture conditions. Female SD rat ASCs from the peri-ovarian, peri-renal, and mesenteric visceral fat could differentiate in vitro into adipocytes, osteocytes, and chondrocytes.

#### 3.4.1. Depot-Specific Differences in Adipogenic Potential

**Figure 4:**
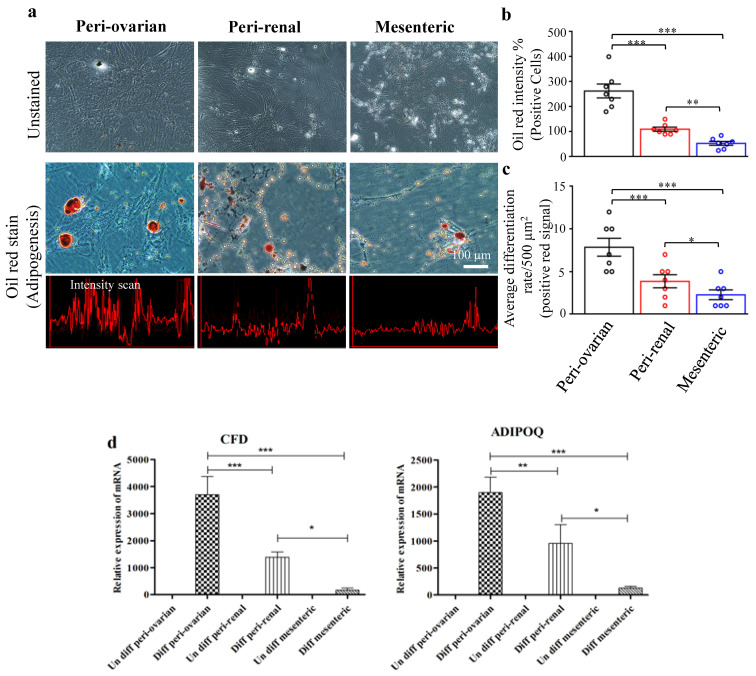
Adipogenic differentiation of ASCs. Light microscopy photographs showed that ASCs at the third passage differentiated into adipocyte lineages. (**a**) Adipogenic differentiation of ASCs from different harvesting sites (peri-ovarian, peri-renal, and mesenteric adipose tissue). Scale bar represents 100 μm. (**b**) Quantitative analysis of Oil red O staining intensity of ASCs. (**c**) The average differentiation rate of ASCs from various harvesting sites. (**d**) RT-qPCR evaluation of adipogenic specific genes, Complement factor D (*CFD*), and Adiponectin (*ADIPOQ*) from various harvesting sites. Values are presented as the mean ± SD with the one-way analysis of variance (ANOVA) followed by Tukey’s post hoc test * *p* < 0.05, ** *p* < 0.01, *** *p* < 0.001. The shown data are representative data from four independent experiments.

Oil Red O staining and expression level of adipocytic markers (*CFD* and *ADIPOQ*) in ASCs from different depots were determined (Figure 4). Although all cell populations formed lipid-filled adipocytes, significant variations in the adipogenic responses were observed among cells from various depots. Adipocyte formation was most extensive and significant in ASCs isolated from the peri-ovarian site (Figure 4). The peri-ovarian ASCs culture had the highest lipid-droplet formation, followed by the peri-renal ASCs culture and mesenteric (Figure 4a–c). These staining signals were specific where no lipid droplet formation was observed in control cultures (Figure 4).

Upon adipocyte induction for eight days, the three cell populations upregulated adipocytic gene markers expression. Higher expression of adipogenic-related genes (*CFD* and *ADIPOQ*) was observed in ASCs cultures of the peri-ovarian site compared to the other anatomical sites, suggesting a better adipogenic differentiation of ASCs isolated from the peri-ovarian site (Figure 4d).

#### 3.4.2. Depot-Specific Differences in Osteogenic Potential

**Figure 5:**
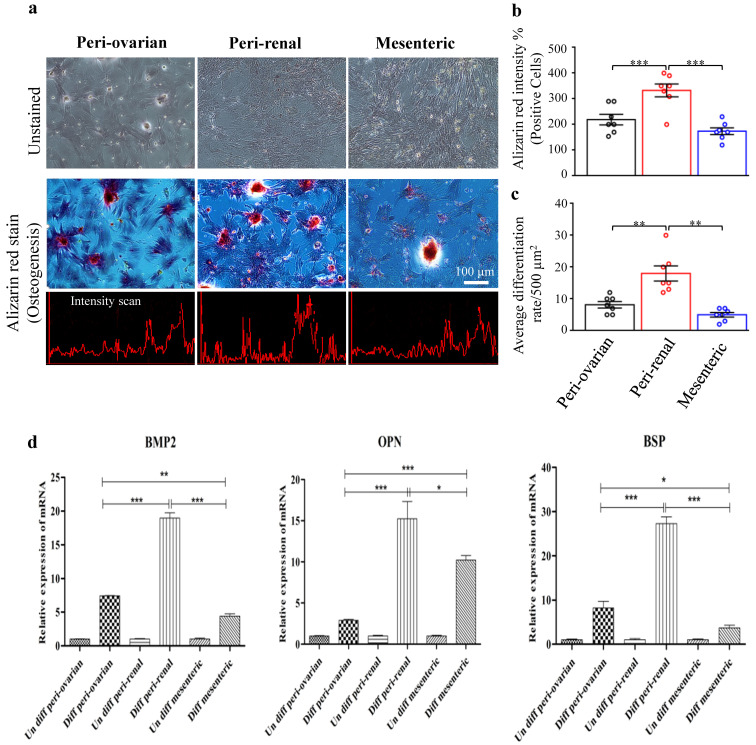
Osteogenic differentiation of ASCs. Light microscopy photographs showed that ASCs at the third passage differentiated into osteocyte lineages. (**a**) Osteogenic differentiation of ASCs from different harvesting sites, peri-ovarian, peri-renal, and mesenteric adipose tissue. Scale bar represents 100 μm. (**b**) Quantitative analysis of Alizarin red staining intensity of ASCs. (**c**) The average differentiation rate of ASCs from various harvesting sites. (**d**) RT-qPCR evaluation of osteogenic specific genes, Bone Morphogenetic Protein 2 (*BMP2*), Osteopontin (*OPN*), and Bone Sialoprotein (*BSP*) from the various harvesting sites. Values are presented as the mean ± SD with the one-way analysis of variance (ANOVA) followed by Tukey’s post hoc test * p < 0.05, ** *p* < 0.01, *** *p* < 0.001. The shown data are representative of four independent experiments.

Adding well-known osteogenic agents, including ascorbic acid, *β*-glycerophosphate, and dexamethasone to the culture media induced osteogenic differentiation in rat ASCs. After 21 days of osteogenic culture, ALZ staining of ASCs revealed the osteogenic development through increased matrix mineralization along culturing time. Cells proliferated quickly, clumped together, and formed dense colonies within two weeks in the osteogenic medium. By comparing ALZ staining of ASCs from various sites, ASCs isolated from the peri-renal adipose tissue showed a highly significant ALZ stain intensity and an average differentiation rate compared to other harvesting sites (Figure 5a–c). Control cultures maintained in a medium without osteogenic inducers were negative (Figure 5a–d).

In RT-qPCR, ASCs of peri-renal adipose tissue revealed a significant upregulation in the *BMP2*, *OPN*, and *BSP* gene expression (Figure 5d). A significant difference was detected in ASCs isolated from peri-ovarian and mesenteric adipose tissue.

#### 3.4.3. Depot-Specific Differences in Chondrogenic Potential

**Figure 6:**
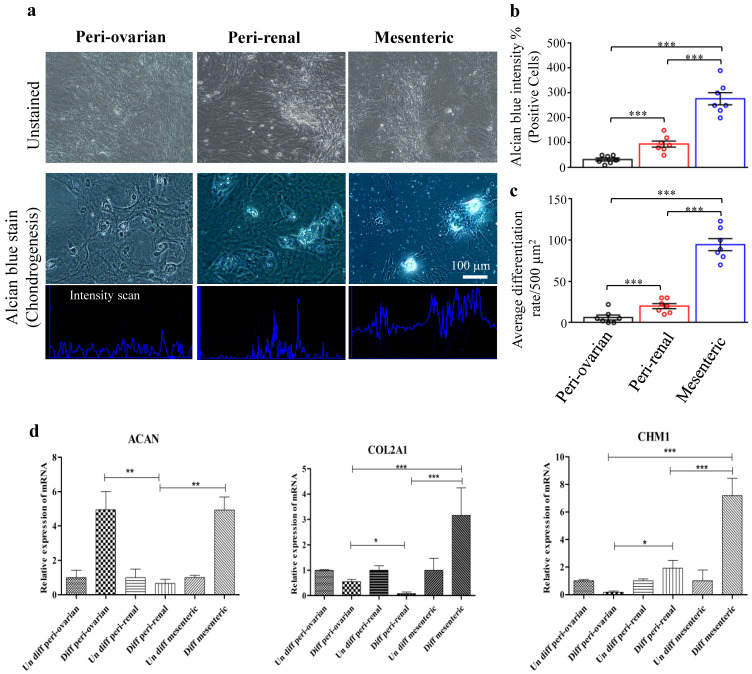
Chondrogenic differentiation of ASCs. Light microscopy photographs showed that ASCs at the third passage differentiated into chondrogenic lineages. (**a**) Chondrogenic differentiation of ASCs from different harvesting sites, peri-ovarian, peri-renal, and mesenteric adipose tissue. (**b**) Quantitative analysis of Alcian blue staining intensity of ASCs. (**c**) Average differentiation rate of ASCs from various harvesting sites. (**d**) RT-qPCR evaluation of chondrogenic specific genes, Aggrecan (*ACAN*), Collagen type 2 (*COL2A1*), Chondromodulin-1 (*CHM1*) from various harvesting sites. Values are presented as the mean ± SD with the one-way analysis of variance (ANOVA) followed by Tukey’s post hoc test * *p <* 0.05, ** *p* < 0.01, *** *p* < 0.001. The shown data are representative of four independent experiments.

Complete chondrogenic media supplemented with well-known chondrogenic agents stimulated chondrogenic differentiation of ASCs. The positive Alcian blue staining of cartilage matrix components indicated ASCs’ ability in chondrogenic lineage differentiation. Cells showed round morphology, low cell density, and extensive cartilaginous matrix deposition. Figure 6a–c shows that ASCs isolated from mesenteric adipose tissue had a highly significant alcian blue staining intensity in addition to a high differential ratio compared to the other anatomical sites. As a control, cultures kept in a medium without chondrogenic inducers were negative for the alcian blue staining.

We further confirmed the chondrogenic capacity by RT-qPCR analysis of the specific chondrogenic markers expression, *ACAN*, *COL2A1*, and *CHM1*. ASCs isolated from the mesenteric adipose tissue showed highly significant *COL2A1* and *CHM1* overexpression compared to the other anatomical sites. At the same time, there was a non-significant difference between the peri-ovarian and mesenteric ASCs in *ACAN* gene expression (Figure 6d).

## 4. Discussion

ASCs have recently received much attention as a potential stem-cell source for regenerative therapeutic applications. Additionally, MSCs derived from rat adult tissues are a prospective source of cells for various cellular therapies. Thus, researchers are keen to learn more about the mechanisms behind their proliferation, differentiation, and heterogeneity [41].

This study designed a side-by-side comparison of three populations of ASCs isolated from adipose tissue of different anatomical sites in female S.D. rats concerning the immune-phenotypic characterization, pluripotency, and differentiation potential of the ASCs population. To our knowledge, this is the first study revealing depot-specific differences in the in vitro osteoblast, adipocyte, and chondrocyte differentiation potential of ASCs isolated from female S.D. rats of peri-ovarian, peri-renal, and mesenteric visceral depots. However, subcutaneous and visceral fat tissues are located differently and have dissimilar metabolic and growth rates [42]. Several adipose depot sites are distributed around the rat body that can be used to isolate ASCs. In this study, we selected the peri-ovarian, peri-renal, and mesenteric visceral adipose tissue of female S.D. rats because these sites are less frequently studied in the literature. Our findings reveal that rat ASCs from these various visceral sites were successfully isolated and expanded.

ASCs from the peri-ovarian, peri-renal, and mesenteric adipose tissue of rats were homogenous in cultures with typical fibroblast-like MSCs. These cells could be easily expanded in vitro without any special requirements under standard culture conditions, consistent with previous reports [43].

MSCs-specific cell-surface markers (CD90, CD44, and CD29) are well documented in rodent MSCs and serve as identifiers of these types of stem cells [44,45,46,47]. Our results show that ASCs at the third passage isolated from different anatomically sites shared a similar immunophenotype expression of CD90, CD44, and CD29 with a non-significant difference. These results are in agreement with the results of Kaewkhaw et al. [33]. We detected minor contaminating hematopoietic cells indicated by the positive CD45 expression, similar to Schäffler and Büchler [48], where they mentioned that ASCs lose the expression of hematopoietic markers with several passages.

A significant debate exists regarding the expression of pluripotency markers in ASCs isolated from rat adipose tissue [49]. The expression of *Oct-4*, *Sox-2*, and *Nanog* transcription factors is critical in controlling self-renewal and differentiation in embryonic stem (E.S.) cells. Their molecular foundation of proliferation and multipotency is well established but with conflicting results [50]. Here, we investigated whether the transcription factors, *Oct-4*, *Sox-2*, and *Nanog*, were expressed in the third passage ASCs isolated from rat visceral fat and if their expression affects these cells’ proliferative and differentiation properties. Similar to Gao et al. [51], our results indicate that *Oct-4*, *Sox-2*, and *Nanog* genes were not expressed in rat ASCs at the third passage isolated from visceral peri-ovarian, peri-renal, and mesenteric fat depots. Additionally, these results were consistent with human studies that found that *Nanog*, but not *Oct-4* and *Sox-2*, is expressed in cultured human adult MSCs [50].

On the contrary, Casella et al. [52] detected a significant expression of stemness marker genes, *Oct4*, *Sox2*, and *Nanog*, in rat ASCs. In this study, ASCs from the three harvesting sites expressed *Rex-1* and *Tert* pluripotency markers. *Rex1* is now widely used as a stem cell marker and plays a crucial role in E.S. cell differentiation [53]. Additionally, *Tert* improves cell proliferation of primary cells and enhances reprogramming efficiency into the induced pluripotent stem cell [54].

Stem cells not only can be kept undifferentiated but also can proliferate and differentiate toward multiple cell lineages. The most popular approaches for evaluating the examined cell populations’ multipotency are assessing their ability to differentiate into adipogenic, osteogenic, and chondrogenic multilineage. Our data show that ASCs from the visceral fat of S.D. rats could also differentiate into adipocyte, osteocyte, and chondrocyte, similar to MSCs isolated from various tissues, consistent with Tholpady et al. [55]. At the same time, considerable variation was evident in the differentiation potential. RT-qPCR results are consistent with the morphology and cell-type-specific staining in assessing the differentiation potential. These results clearly illustrate that ASCs from various adipose depots have different capacities to initially respond to osteogenic, adipogenic, and chondrogenic media.

Differentiation capacity is the key issue for the clinical translation of stem cells to tissue engineering. Our results demonstrated that ASCs adipogenic, osteogenic, and chondrogenic differentiation potential varied with the fat depots regardless of cell morphology and immunophenotype similarity. The heterogeneity of adipose tissue might nicely explain our findings as adipose tissues are composed of adipocytes and SVF containing different cell types. In addition, the adipose tissue is an endocrine tissue since both adipocytes and SVF cells secrete several cytokines revealing different biological consequences [56]. Therefore, individual fat depots exhibit unique profiles of adipokines and interleukin secretion. Analyzing rat visceral fat adipose tissue (perinodal adipose tissue around lymph nodes, perivascular adipose tissue, and pericardial adipose tissue) demonstrated different site-specific properties of adipose tissue and paracrine interactions among minor adipose depots and adjacent tissues [57].

Studies on human adipose tissue have shown that adipose tissues have different characters according to their anatomic locations, and individual fat depots exhibit distinct embryonic origins and express different *HOX* code (*HOX* gene expression profile) [58,59]. These homeotic genes (*HOX*) are transcription factors playing a critical role in the cell positioning during embryonic development, driving the differentiation of tissue stem cells towards their respective lineages to repair and maintain the correct function of tissues and organs, mature cells regulation in adults, and stem cell positional identity of fat cells [58,60]. Foissac et al., 2017 [58] suggested that breast fat has an embryogenic origin distinct from the knee and thigh fat. In addition, Kouidhi et al., 2015 [59] revealed that chin and knee fat depots expressed a different *HOX* code and could have different embryonic origins. Matching the embryonic origin and *HOX* code between the host tissue and the donor fat sites is essential to improve the safety and postoperative outcomes of autologous fat grafting [56]. Together, these studies indicate that the embryonic origin of ASCs varies according to fat depots and could impact the differentiation capacity and guide the donor site for fat grafting.

Of note, the differentiation potential variability between the different studied harvesting sites may also be explained by the presence of a lineage “imprinting” in separate stromal cell compartments that influence the differentiation potential of MSC, as previously illustrated [61,62]. Adipose tissue is a highly heterogeneous tissue between individuals and when comparing different fat depots within one individual. The differences between fat depots have been demonstrated regarding the metabolic response of adipose tissue to various hormonal and neurological effects [63]. Lineage imprinting has also been observed at early hematopoietic progenitors [64] and during placental development [63]. A recent study revealed an evolutionary mechanism whereby maternally silenced genes arise from biallelically expressed progenitors [65].

ASCs isolated from peri-ovarian fat showed the highest adipogenesis differentiation potential with the most intense Oil Red staining. These results were confirmed with significant upregulation of *CFD* and *ADIPOQ* gene marker expression by RT-qPCR. These results could be explained by the presence of sex hormones which play an essential role in regulating the differentiation and distribution of adipocytes. Still, the underlying mechanisms have not been fully elucidated [57,66].

The high intensity of the ALZ stain plus the highest expression of the osteogenesis-related gene (*BMP2*, *OPN*, and *BSP*) were observed in ASCs cultures of the peri-renal site compared to the other anatomical sites suggesting a better osteogenic differentiation of ASCs isolated from the peri-renal site. The peri-renal adipose tissue is an atypical visceral fat pad with a complete blood supply system, lymph fluid drainage, and innervation near the kidneys [67]. Local renin-angiotensin activity is high in peri-renal adipose tissue [68], influencing adipocyte development and differentiation [69]. Therefore, it is possible that hormonal factors are to account for the peri-renal ASCs’ high osteogenic potential found in this study. On the other hand, the osteogenic differentiation of ASCs from peri-renal fat could be linked to the complex interaction between kidneys and bone, as well as the role of kidneys in the regulation of bone formation and metabolism, as well as the maintenance of calcium and phosphorus homeostasis, which is required for bone mineralization and development. Additionally, several chemicals produced by the kidneys, such as 1,25(OH)_2_D_3_, Klotho, bone morphogenetic protein-7, and erythropoietin, are involved in bone formation, remodeling and repair [70].

Additionally, the Alcian blue staining and the expression of chondrogenic specific genes (*ACAN*, *COL2A1*, and *CHM1*) had the highest values in ASCs isolated from the mesenteric fat, indicating their higher potential to differentiate into chondrogenic tissue than the other anatomical sites.

Although rats tested in this study were apparently lean and future research is required to address the effect of fat depot sites on ASCs differentiation potential in obese rats, our results provide valuable information for regenerative therapy research.

## 5. Conclusions

The cell source should be chosen based on the clinical application goal. Identifying compartment-specific MSC molecular signatures in vitro can aid in the development of a set of molecular markers that are predictive of MSC in vivo biological behavior and can be used to screen cultured MSCs before clinical use. Herein, the analysis of ASCs from different depots suggests the superiority of peri-ovarian visceral fat ASCs for adipogenesis, in addition to the more substantial capability of peri-renal ASCs for osteogenesis. However, mesenteric ASCs fat showed a better chondrogenic differentiation potential.

## Figures and Tables

**Table 1:** Gene primers used for qPCR.

Name	Direction	Primer Sequence (5′—3′)	Refrence
*ADIPOQ*	Forward Reverse	TAATTCAGAGCAGCCCGTAGTGGGGATAACACTCAGAACC	[36]
*CFD*	Forward Reverse	GGAGTGACCAAGGATGAGG ACCCAGTGAGGCATTGTG	[36]
*BMP2*	Forward Reverse	CAGGTCTTTGCACCAAGATG GCTGGACTTAAGACGCTTCC	[37]
*OPN*	Forward Reverse	GAAGAGCCAGGAGTCCGATG CTTCCCGTTGCTGTCCTGAT	This study (M14656.1)
*BSP*	Forward Reverse	AGGCTACGAGGGTCAGGATT GCACCTTCCTGAGTTGAGCT	This study (XM_017599076.2)
*ACAN*	Forward Reverse	CTCTGCCTCCCGTGAAAC TGAAGTGCCTGCATCTATGT	[38]
*COL2A1*	Forward Reverse	TCCTAAGGGTGCCAATGGTGA AGGACCAACTTTGCCTTGAGGAC	[39]
*CHM1*	Forward Reverse	GAGAACTGTGAGGGCTGTCA GATACCTCGGGCCAGAAGTG	This study (NM_030854.1)
*β-actin*	Forward Reverse	GCAGGAGTACGATGAGTCCG ACGCAGCTCAGTAACAGTCC	[40]

## Data Availability

Data are contained within the article.
